# Assessing health insurance literacy in Switzerland: first results from a measurement tool

**DOI:** 10.1093/eurpub/ckad190

**Published:** 2023-10-23

**Authors:** Tess L C Bardy

**Affiliations:** Faculty of Health Sciences and Medicine and Center for Health, Policy and Economics, University of Lucerne, Alpenquai 4, Lucerne, 6005 Switzerland

## Abstract

**Background:**

Health insurance literacy (HIL) is crucial for individuals to make informed-decisions and navigate complex choice-based health insurance systems. However, there is a lack of evidence on HIL in countries outside the US, with Switzerland no exception.

**Methods:**

Using the HILM-CH, a survey instrument developed to measure HIL in Switzerland, this study first describes the answers to the HILM-CH. Second, the study uses ordinary least squares and quantile regressions to investigate the associations between the HIL score and demographic, socioeconomic, health, and preference factors in the German, French, and Italian Swiss regions.

**Results:**

A third of the population faces difficulties in finding health insurance information. Understanding it and managing the financial aspects of the Swiss health insurance system pose the biggest barriers to the population. The HIL score significantly and positively correlates with age and financial risk, while non-Swiss individuals have lower HIL scores. No association was found between HIL, gender, education and time preference. There is a small health gradient, with more doctor visits associated with higher HIL in the lowest quantiles of the HIL score. Similarly, wealthier individuals in the Swiss German part of Switzerland have a higher HIL when choosing their health insurance.

**Conclusion:**

This study provides important insights into Swiss HIL and its associated factors. These findings contribute to the international literature on HIL and highlight the importance of understanding variations in HIL and various factors in choice-based health insurance systems.

## Introduction

Health insurance literacy (HIL) has been discussed as a critical lever to ensure informed choices and efficient use of health insurance plans in choice-based health insurance systems.^1^ HIL is a concept that encompasses individuals’ ability to seek, understand, evaluate and further use information related to health insurance.^2^ Thus, HIL may explain the barriers, such as the incapacity to understand health insurance plans’ terms, health, and financial implications that prevent consumers from making informed choices and efficiently using their health insurance plan.^1^^,3^ Problems resulting from low HIL include over- or under-insurance, delayed care and financial burden.^1^^,2,^^4^

Previous studies in the United States (US) have examined associations between HIL levels and population groups to support their health insurance navigation.^4–6^ They found that low-educated, young individuals, migrants, or people who have little experience with the healthcare system had the lowest HIL. Their limited ability to make informed choices and effectively use their health insurance was associated with a higher likelihood of foregoing needed care and facing financial burdens.

This study enhances the generalizability of these results beyond US samples by investigating the context of Switzerland’s choice-based health insurance system.^7^^,^^8^ Prior studies have shown Swiss consumers’ limited ability to navigate health insurance, with a significant part of the population not knowing where to find health insurance information or being unaware of their health insurance plan details.^9^ This comes vis-à-vis the Swiss highest out-of-pocket healthcare expenditures and a significant part of the population skipping medical care due to costs.^10^ Therefore, to reduce these health and financial burdens, it is especially important to understand the barriers to navigating health insurance in Switzerland and which population groups have lower HIL.

Switzerland’s health insurance system mandates individuals to purchase basic health insurance from a selection of plans regulated by the Federal government. Insurance premiums are community-rated and vary across the 50 health insurers, primarily based on cost-sharing, plan type and age groups. Individuals can choose a yearly deductible ranging from 300 to 2500 Swiss francs. After reaching the deductible, they are responsible for a 10% copayment rate, capped at 700 Swiss francs per year. Additionally, individuals must choose between two plan types: unrestricted and restricted healthcare provider access. The latter, known as managed care, includes telemedicine, health maintenance organization (HMO) and family doctor health plans. Moreover, individuals have the flexibility to select their health insurer based on factors like reputation and ease of claims processing.^11^

In this context, this study builds upon the recent validation of the Swiss Health Insurance Literacy Measure (HILM-CH), a survey instrument that measures self-perceived barriers when choosing and using health insurance,^8^ to assess HIL in Switzerland. The primary objectives of this study are two-fold. First, it identifies perceived barriers to navigating health insurance. Second, it investigates the associations between HIL and demographic, socioeconomic, health and preference factors (specifically, financial risk aversion and time preference) using ordinary least squares (OLS) and quantile regressions, recognizing that the relationship between these factors and HIL may vary along the HIL distribution. The study explores region-specific variations in HIL across German, French and Italian-speaking regions of Switzerland, acknowledging the potential influence of cultural differences on health insurance behaviour, similar to their impact on job market dynamics, social insurance and political preferences.^12–15^ Understanding the region-specific variations is essential in light of developing targeted interventions to support health insurance navigation in Switzerland.

This study contributes to the literature in various ways. First, it represents the first measure of HIL and its variations across population groups in Switzerland. Second, the findings will provide valuable insights for evidence-based policy decisions to improve HIL and promote more efficient use of healthcare resources in Switzerland, aligning with the general literature on health and healthcare in Switzerland, as documented in the Swiss Health Care Atlas.^16^ Third, the study sheds light on Switzerland’s cultural differences by examining region-specific variations in HIL, providing valuable insights into each region’s influence on health insurance decisions. Lastly, the study advances the HIL international literature by exploring factors associated with HIL in a novel way using quantile regressions,^4^^,^^6^^,^^7^ extending examined factors with preference factors for deeper insights into HIL variations.^7^,^17^

## Methods

### Data

#### Survey and sample

The data comes from the 2021 Swiss Health Insurance Literacy Survey administered by the University of Lucerne. This representative online survey gathered 6036 participants aged between 26 and 75, living in Switzerland’s German-, French-, and Italian-speaking parts. The data collection was outsourced to intervista AG, a private market research company under the General Data Protection Law and the Federal Act on Data Protection in Switzerland. No ethical approval was needed for the study. The survey included questions about respondents’ health insurance and sociodemographic background. The questionnaire was available in German, French and Italian. Representativeness was ensured across the linguistic regions using quota sampling based on education, gender and age. Sample weights are used to correct for over- and under-representation.

#### HILM-CH

The HILM-CH is a survey instrument designed to assess HIL in the Swiss population. It consists of 21 items that measure HIL across four scales (or domains):

- Selecting a health insurance
  - Scale 1: Confidence in choosing health insurance
  - Scale 2: Comparing health insurance plans
- Using a health insurance plan
  - Scale 1: Confidence in using health insurance
  - Scale 2: Proactive use of health insurance

Each item that composes the HILM-CH can be answered from 1 to 4, 1 being ‘not confident/not likely at all’ and four being ‘extremely confident/likely’. See Bardy^8^ for more information about the psychometric properties and cross-cultural validation of the HILM-CH. Respondents’ answers to each item were averaged to create a HIL score. Similarly, four scale scores can be created. A lower score indicates low HIL and thus higher barriers to navigate health insurance.

#### Selected factors

This study examines HIL and various factors. Demographic include gender, age and nationality. Socioeconomic factors include education and monthly income in Swiss Francs, and the number of doctor visits in the past 12 months proxies the health status. Preference factors involve measures of financial risk aversion and time preference. Financial risk aversion is measured using: ‘Would you consider yourself a person who is fully prepared to take risks, or do you try to avoid risk?’.^18^ Time preference is measured using: ‘Would you consider yourself a person who is fully prepared to give up something today and benefit from it in the future?’.^19^ Both items are rated on a scale from 1 to 5, with one indicating ‘not prepared at all’ and five indicating ‘fully prepared’.

Previous research^5^ suggests an age gradient, with older individuals reporting higher HIL due to more experience, while non-Swiss individuals may have lower HIL due to language or system barriers. Higher socioeconomic status is expected to be associated with higher HIL, as educated individuals are more knowledgeable, and higher incomes may acquire health insurance knowledge independently.^20^ More doctor visits might relate to higher HIL as individuals become more familiar with the system.^5^ Preference variables could be critical in selecting and utilizing health insurance plans, as risk-averse individuals are informed about health insurance to avoid risk.^17^^,^^21^^,^^22^ Similarly, more future-oriented individuals may have greater confidence in their knowledge of health insurance decisions, making them more likely to be aware of health insurance details.^23^

### Statistical analysis

Descriptive statistics were reported for the HIL score and each of the HILM-CH four scales. Associations analyses were performed using OLS (‘reg’ command on STATA 17.1) and quantile regressions (using ‘qreg’), with the HIL score being the dependent variable. Based on the hypothesis that the relationships between the HIL score and the potential associated factors defined in the previous section may differ in intensity along with the HIL score distribution, quantile regressions were carried out for Q25, Q50, Q75 and Q90. Other selected factors are included as controls.

Quantile regressions are analysed using trend charts reporting the marginal effects of the standardised quantile regression estimates for each selected variable and conditional on each quantile of the HIL score. Marginal effects were drawn on standardised estimates obtained from the quantile regressions. An association was considered statistically significant if the 95% confidence interval did not overlap with the horizontal line corresponding to zero coefficient estimates.

## Results

### Sample description

Table 1 presents the sample characteristics for the total HILM-CH score per domain. Of the 6036 respondents, the majority are from the German-speaking area of Switzerland, followed by the French- and Italian-speaking regions. The gender and age distributions are comparable to official statistics reported by the Swiss Federal Statistical Office,^24^ and about a third of the sample completed tertiary education. The median income is 6000 Swiss francs per month. The average number of visits to a doctor within the past 12 months is four. Furthermore, respondents demonstrate a tendency towards financial risk aversion (mean = 2.39, SD = 1.20) and are forward-looking (mean = 3.4, SD = 1.16).

**Table 1:** Weighted sample descriptive statistics and HILM-CH scores distribution

		Sample (*N* = 6036)		Total score		Selecting health plans				Using health plans			
						Confidence in choosing		Comparing plans		Confidence in using		Being proactive	
				2.86	0.52	2.86	0.56	2.82	0.63	2.74	0.67	3.01	0.63
		*N*	%	Mean	SD	Mean	SD	Mean	SD	Mean	SD	Mean	SD
Demographic characteristics	**Age** (Mean, SD)	49.75	13.76	2.86	(0.52)	2.86	(0.56)	2.82	(0.63)	2.74	(0.67)	3.01	(0.63)
	**Gender**												
	Male	3016	49.97	2.85	(0.52)	2.82	(0.63)	2.88	(0.56)	2.73	(0.67)	2.95	(0.62)
	Female	3020	50.03	2.87	(0.53)	2.82	(0.64)	2.85	(0.56)	2.75	(0.68)	3.06	(0.63)
	**Nationality**												
	Swiss	5503	91.17	2.87	(0.52)	2.83	(0.63)	2.87	(0.56)	2.76	(0.66)	3.02	(0.62)
	Non-Swiss	533	8.83	2.74	(0.56)	2.71	(0.67)	2.77	(0.58)	2.59	(0.72)	2.90	(0.64)
Socioeconomic characteristics	**Education**												
	Primary/secondary	3846	63.72	2.86	(0.52)	2.81	(0.63)	2.76	(0.67)	2.76	(0.67)	3.01	(0.63)
	Tertiary	2190	36.28	2.86	(0.52)	2.84	(0.63)	2.72	(0.68)	2.72	(0.68)	3.00	(0.61)
	**Monthly income (in Swiss francs)**												
	Less than 4500	1883	31.2	2.85	(0.54)	2.83	(0.64)	2.79	(0.64)	2.75	(0.69)	3.01	(0.65)
	4501–6000	1528	25.32	2.85	(0.52)	2.86	(0.55)	2.81	(0.62)	2.72	(0.66)	3.00	(0.62)
	6001–9000	927	15.35	2.89	(0.51)	2.91	(0.55)	2.86	(0.63)	2.75	(0.67)	3.03	(0.61)
	More than 9000	1698	28.13	2.87	(0.51)	2.88	(0.55)	2.84	(0.63)	2.76	(0.66)	2.99	(0.62)
Health and preferences	**Doctor visits** (Mean, SD)	3.93	5.30	2.86	(0.52)	2.86	(0.56)	2.82	(0.63)	2.74	(0.67)	3.00	(0.63)
	**Risk preference** (Mean, SD)	2.39	1.20	2.86	(0.52)	2.86	(0.56)	2.82	(0.63)	2.74	(0.67)	3.00	(0.63)
	**Time preference** (Mean, SD)	3.40	1.16	2.86	(0.52)	2.86	(0.56)	2.82	(0.63)	2.74	(0.67)	3.00	(0.63)
	**Language**												
	German	4274	70.81	2.90	(0.52)	2.88	(0.56)	2.87	(0.63)	2.79	(0.68)	3.05	(0.63)
	French	1455	24.11	2.77	(0.51)	2.83	(0.56)	2.69	(0.63)	2.65	(0.64)	2.90	(0.62)
	Italian	678	5.08	2.77	(0.51)	2.79	(0.57)	2.76	(0.63)	2.62	(0.66)	2.90	(0.60)

*Source*: Swiss Health Insurance Literacy Survey 2021. *Note*: Reported numbers depict the weighted characteristics of the total sample and averaged scale score. Education: tertiary education refers to respondents who obtained a degree from a University of Applied Sciences (‘Höhere Fach- oder Berufsausbildung’ and ‘Fachhochschule’), a college of education (‘Pädagogische Hochschule’), a Swiss University, or a Swiss Federal Institute of Technology (‘Eidgenössische Technische Hochschule’). All other (un)completed education levels were assigned to primary and secondary education. Doctor visits: number of doctor visits in the last 12 months.

The average HIL score is 2.86 (SD = 0.52). Non-Swiss respondents have the lowest HIL score (2.74, SD = 0.56), and respondents aged 65 or more displayed the highest one (2.94, SD = 0.52). When looking at the four domain scores, ‘being proactive’ has the highest score (3.01, SD = 0.63), and ‘confidence in using health plans’ is the lowest one (2.74, SD = 0.67).

### Identifying barriers

**Figure 1:**
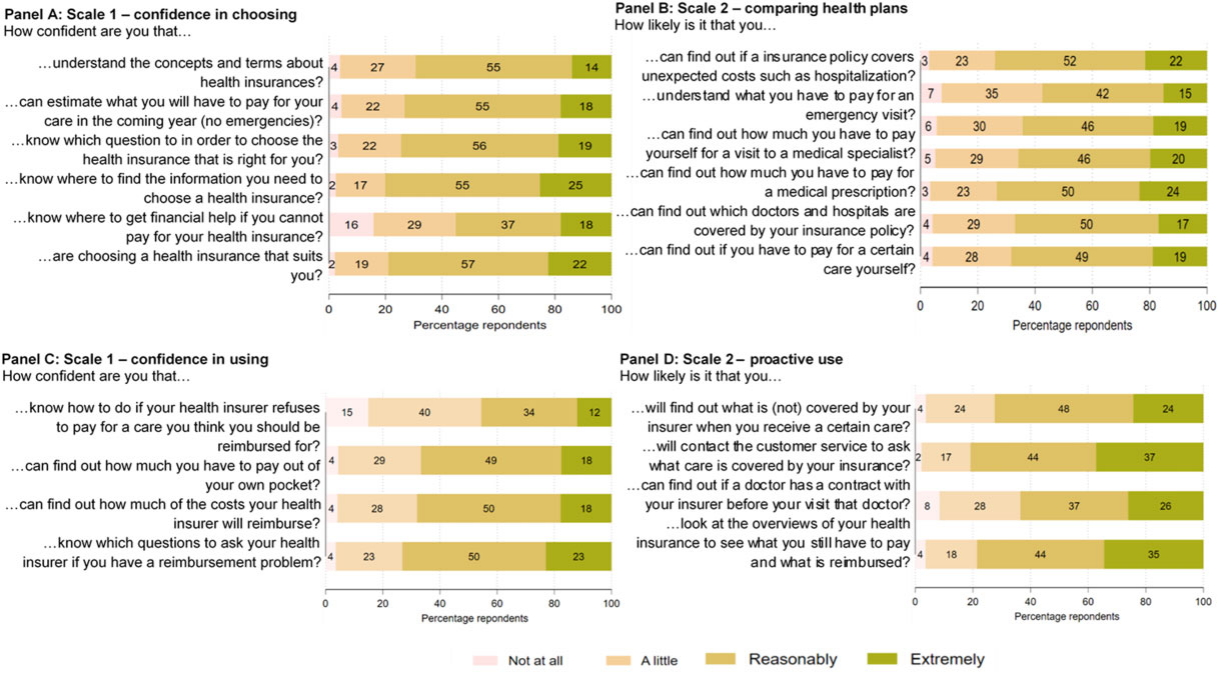
Description of the HILM-CH items per scale

Figure 1 describes the HILM-CH results per item in the four domains, respectively, confidence in choosing health plans (Panel A), comparing health plans (Panel B), confidence in using a health plan (Panel C), and being proactive in using a health plan (Panel C).

When choosing a health insurance plan, a third of the sample reports not being able to understand the concepts and terms of health insurance, and more than 40% of the respondents are little or not confident at all about knowing where to get financial help if they cannot pay for their health insurance. Finding information is a perceived barrier for a third of the population (Panel B), and more than 40% of the respondents do not know how much to pay to visit the emergency room.

When looking at the use of health insurance (Panels C and D), while more than half of the sample is reasonably or extremely confident regarding the use of their health insurance plan, close to 60% of the sample does not know what to do if the health insurer refuses to pay for certain care that they think they should get reimbursed. Finally, while about 36% of the sample is unlikely to find out whether a doctor has a contract with their health insurer and therefore avoids extra costs, between 60 and 80% of the sample is proactive when using their health insurance (Panel D).

### HIL associations

#### OLS regressions outputs

Table 2 reports the region-specific associations between HIL score and selected factors using OLS regressions. Estimates for each of the four subscales can be found in Supplementary table A1. These associations should not be interpreted as causal relationships.

**Table 2:** Multiple linear regression estimates for the HIL score

	German	French	Italian
Female	0.0492 (0.0171)	0.0334 (0.0279)	0.0276 (0.0432)
Age	0.00337 (0.000616)	0.00321 (0.00104)	0.00196 (0.00154)
Non-Swiss	−0.0482 (0.0377)	−0.0809* (0.0462)	−0.106* (0.0595)
Monthly income in CHF (base: <4500)			
4500–5999	0.0136 (0.0231)	−0.0216 (0.0355)	0.0165 (0.0596)
6000–8999	0.0685 (0.0268)	0.0103 (0.0413)	0.0640 (0.0717)
≥9000	0.0419* (0.0221)	−0.0407 (0.0367)	0.0143 (0.0513)
Tertiary education	0.0253 (0.0177)	−0.0143 (0.0294)	0.0286 (0.0484)
Number of doctor visits	0.00570 (0.00171)	0.00587 (0.00227)	−0.00464 (0.00383)
Financial risk	0.0440 (0.00824)	0.0520 (0.0124)	0.0536 (0.0186)
Time preferences	−0.00767 (0.00871)	−0.000273 (0.0118)	−0.0465 (0.0218)
Constant	2.556 (0.0521)	2.321 (0.142)	2.587 (0.146)

Observations	3903	1455	678
*R*-squared	0.030	0.046	0.130
Adjusted *R*^2^	0.0211	0.0291	0.0895

*Source*: Swiss Health Insurance Literacy Survey 2021. *Notes*: The table shows the estimated coefficients from linear regressions per language groups. Robust standard errors in parentheses. Other controls included: 26 canton dummies.

*** *P* < 0.01, ** *P* < 0.05, * *P* <0.1.

The findings suggest that females in the German-speaking region of Switzerland report a significantly higher HIL score than males. Upon closer inspection of Supplementary table A1, this result is driven by women being more proactive when using their health insurance plans than men; it is possible to observe the same pattern in the French and Italian groups, although this result vanishes away in the pooled OLS regressions. On average, non-Swiss individuals in the French and Italian regions have a significantly lower HIL than Swiss individuals.

Results further indicate age and health gradients. Older individuals and individuals who have had more doctor visits in the last 12 months than their counterparts exhibit higher HIL scores in the German- and French-speaking regions, specifically on scales 2–4 (see Supplementary table A1). However, the magnitude of the effect remains low.

There is no significant relationship between tertiary education and HIL score compared to low/mid-education levels. Moreover, only wealthier German-speaking respondents reported a significantly higher HIL score than the baseline. This result is driven by higher scores in the first two scales of the HILM-CH ‘Selecting health insurance’ (see Supplementary table A1). Lastly, individuals with lower financial risk aversion reported a higher HIL score in all three linguistic regions.

#### Quantile regressions and marginal effect estimates

**Figure 2:**
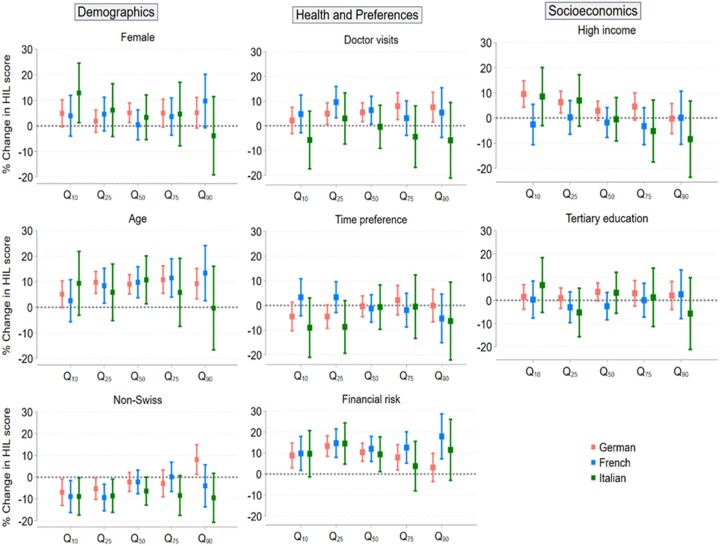
Marginal effects of selected factors on each conditional HIL score quantile per linguistic region

Figure 2 complements the OLS analyses with marginal effects of selected factors on the total HIL score per linguistic region, conditioned on the 10th, 25th, 50th, 75th and 90th quantiles of the HIL score distribution.

In the first column, although not statistically significant, women tend to have higher HIL scores than men in all three linguistic regions of Switzerland. Similar to OLS outputs, an age gradient is detected in the German and French regions, where HIL scores are approximately 10% higher for older individuals than their younger counterparts. Similarly, non-Swiss individuals exhibit significantly lower HIL scores in the lowest quantiles of the HIL score distribution compared to Swiss individuals. This significant difference vanishes with higher quantiles in all three language regions, except for the 90th quantile in the German region, where non-Swiss individuals have an average HIL score significantly higher by almost 10% compared to Swiss individuals.

In the second column, there is a small yet significant effect of the number of doctor visits on the distribution of HIL score. Specifically, individuals who reported more doctor visits tend to have higher HIL scores by around 5% in the Swiss German region (from the 25th to 90th quantiles) and in the French region (from the 25th to 50th quantiles). However, the Italian region has no significant effect due to more variability.

There is no significant change in HIL score regarding future-oriented behaviour individuals. However, substantial variations in HIL scores across the linguistic regions concerning financial risk exist. For instance, risk-seeking individuals in the French-speaking region tend to have higher HIL scores by 5% to 10% compared to their risk-averse counterparts. These findings suggest that risk-seeking behaviour may play a role in influencing HIL scores in certain linguistic regions of Switzerland.

The last column of figure 2 shows no education gradient as hypothesized, suggesting that education level may not be a significant predictor of HIL score in Switzerland. Similarly, no income gradient is found, except for the first two quantiles of the HIL distribution in the Swiss German group, where wealthier individuals exhibit HIL scores between 5% to 10% higher than lower-income individuals. These results suggest that income significantly mitigates a low HIL in the Swiss German group when looking at individuals with the most difficulties navigating the Swiss health insurance system.

## Discussion

The study presents the first assessment of HIL in Switzerland. The study’s primary objectives were to identify perceived barriers faced by the population when navigating health insurance, investigate the associations between HIL and demographic, socioeconomic, health, and preference factors, and shed light on HIL variations across different population groups.

The study identifies various barriers to navigating health insurance in Switzerland—notably understanding and managing health insurance plans’ financial aspects. Further, a third of the population report a barrier in finding information when choosing a health insurance plan, aligning with previous evidence.^9^ This result could point to the information overload that creates barriers in navigating health insurance^18^^,^^25^ and suggests the need for targeted decision aids to support consumers in making informed choices effectively. A relevant example from the US is ‘Show Me My Health Plans’ SMMHP.^26^ Designed to provide education, an annual out-of-pocket cost calculator, along with personalised plan recommendations, the SMMHP significantly improved health insurance knowledge, decision self-efficacy, confidence in health insurance choices, and HIL resulting in better plan selection compared to using general health insurance information. Similar decision aids in Switzerland could help alleviate perceived barriers in navigating health insurance and increase HIL.

Factors associated with HIL were investigated using region-specific OLS and quantile regressions. As in the literature,^4^ both models find an age gradient with older individuals exhibiting higher HIL scores than younger counterparts in the French and German-speaking regions. Because younger individuals are healthier and utilise fewer healthcare services, they may perceive health insurance choices as less critical and, thus, report a lower HIL score. Addressing HIL levels proactively in this younger group can help improve their health insurance decision-making in the future.^27^ Further, non-Swiss individuals display the lowest HIL probably due to the complexity of the Swiss health insurance system and potential language barriers, highlighting the need to support this population group.

Only an income gradient was found in the German-speaking region. This result is driven by the significantly higher scores when choosing health insurance (scales 1 and 2 of Supplementary table A1). This discrepancy in the results could be explained by a higher level of risk aversion among Swiss German individuals,^12^^,^^13^^,^^15^ leading them to seek plans that align with their health and financial needs. Wealthier individuals could thus take advantage of the developed network of health insurance brokers,^28^ an expensive service that chooses the best plan for one’s health and finances but does not intervene in its utilisation (no significant associations observed in scales 3 and 4 of Supplementary table A1).

More doctor visits correlates with the German speakers’ higher HIL scores and the French-speaking region’s low score, reinforcing previous findings.^5^ No association between HIL and time preference was found. However, results depicted that risk-seeking individuals are better off along the whole HIL score distribution than risk-averse individuals. Recent evidence showed that Swiss risk-seeking individuals tend to choose more flexible health insurance plans, involving a certain attitude and knowledge towards choosing their health insurance.^29^ Likewise, Korkmaz *et al*.^30^ underline the association between risk-seeking behaviour and higher financial literacy.

HIL distributional analyses can bring valuable insights into population groups’ needs to target for policy interventions to enhance HIL and support health insurance decision-making. This novel approach opposes two methods proposed in the US literature but that lack justification and whose results vary greatly.^7^ The first method involves grouping respondents who answered ‘not confident/likely at all’ or ‘a little confident/likely’ to at least one item of the HILM-CH as having an inadequate HIL level, while the rest were classified as adequate.^6^ The second approach suggests dividing the HIL score into three equally large groups of HIL: low, intermediate or high. Future research is therefore warranted to propose a single method for classifying and comparing HIL scores internationally.^7^^,^^31^

This study has some limitations to consider. The HILM-CH is a self-perceived measurement tool, potentially leading to discrepancies between individuals’ perceptions and their actual knowledge, warranting further investigation in this area. Further, the survey was administered online, potentially omitting population groups that are not digitally literate. Additionally, the sample used in that study does not consider the population over 75 and individuals aged under 25. Due to different healthcare needs, more research is required to better understand these age groups’ HIL.

Finally, the models’ low R-squared values suggest that selected factors only explain a fraction of the variations in HIL, indicating the need to consider additional factors, such as behavioural factors (or biases, including hassle costs, inattention or optimism bias) influencing health insurance decision-making,^17^ which population groups are subject to these biases, and what interventions can help them. Future research could explore these factors to enhance the understanding of HIL in Switzerland and abroad.

In conclusion, this study contributes valuable evidence to the field of HIL in Switzerland. Its identification of barriers to navigating health insurance and factors associated with low HIL can guide policymakers in developing educational programmes and decision aids (e.g. the SMMHP) to address barriers and improve health insurance decision-making, ultimately enhancing HIL. In the long term, education programmes could introduce health insurance education as part of the elementary and middle school curriculum.^32^ In the shorter term, decision aids with the help of social media campaigns^33^ could offer promising avenues to raise public awareness about health insurance benefits and functioning. The study’s findings provide a foundation for further research to advance HIL understanding and decision-making in Switzerland and other choice-based insurance systems worldwide.

## Supplementary Material

ckad190_Supplementary_Data

## Data Availability

The data that support the findings of this study are available free of charge upon request after signing a data contract with the Center for Health, Policy and Economics (CHPE) at the University of Lucerne, Switzerland. Contact by email via chpe@unilu.ch with a brief description of the planned research and dissemination of results. Restrictions apply to the availability of data that are part of a broader study and provided by intervista AG. Data users may gain access to datasets only after accepting an agreement to use and cite the data in a proper fashion, for scientific research and education within an academic framework, and following typical scientific, ethical norms of conduct. However, all datasets will be available from corresponding author upon reasonable request.
