# Supplementary material for: Assessing health insurance literacy in Switzerland: first results from a measurement tool
Source: Eur J Public Health. 2023 Oct 23;34(2):237–43. doi: 10.1093/eurpub/ckad190 (PMC10990528; doi:10.1093/eurpub/ckad190)
Supplement: ckad190_Supplementary_Data [file ckad190_supplementary_data.docx]

Table A1: multiple linear regressions estimates per language group for each HILM-CH subscale

|  | **German** | | | | |  | | **French** | | | |  | **Italian** | | | |  |
| --- | --- | --- | --- | --- | --- | --- | --- | --- | --- | --- | --- | --- | --- | --- | --- | --- | --- |
| **Scales** | **1** | **2** | **3** | **4** |  | | **1** | | **2** | **3** | **4** |  | **1** | **2** | **3** | **4** |  |
|  |  |  |  |  |  | | |  |  |  |  |  |  |  |  |  |  |
| Female | 0.005 | 0.038* | 0.038* | 0.116*** |  | | | 0.025 | -0.019 | 0.032 | 0.095*** |  | -0.030 | 0.060 | -0.021 | 0.101** |  |
|  | (0.019) | (0.021) | (0.022) | (0.021) |  | | | (0.031) | (0.035) | (0.035) | (0.034) |  | (0.049) | (0.056) | (0.054) | (0.051) |  |
| Age | 0.000 | 0.002*** | 0.004*** | 0.007*** |  | | | 0.001 | 0.003** | 0.005*** | 0.003** |  | 0.001 | 0.002 | 0.001 | 0.004* |  |
|  | (0.001) | (0.001) | (0.001) | (0.001) |  | | | (0.001) | (0.001) | (0.001) | (0.001) |  | (0.002) | (0.002) | (0.002) | (0.002) |  |
| Non-Swiss | -0.060 | -0.052 | -0.070 | -0.011 |  | | | -0.091* | -0.067 | -0.117** | -0.049 |  | -0.135* | -0.066 | -0.155** | -0.068 |  |
|  | (0.039) | (0.043) | (0.048) | (0.042) |  | | | (0.048) | (0.060) | (0.059) | (0.056) |  | (0.069) | (0.077) | (0.074) | (0.070) |  |
| Monthly income in CHF (base: < 4500) |  |  |  |  |  | | |  |  |  |  |  |  |  |  |  |  |
| 4500 - 5999 | 0.030 | 0.032 | -0.003 | -0.006 |  | | | -0.014 | -0.041 | -0.069 | 0.037 |  | 0.017 | 0.009 | 0.016 | 0.023 |  |
|  | (0.025) | (0.027) | (0.030) | (0.027) |  | | | (0.039) | (0.044) | (0.044) | (0.044) |  | (0.067) | (0.075) | (0.075) | (0.068) |  |
| 6000 - 8999 | 0.081*** | 0.098*** | 0.040 | 0.055* |  | | | 0.029 | -0.003 | 0.003 | 0.012 |  | 0.028 | 0.063 | 0.061 | 0.105 |  |
|  | (0.029) | (0.032) | (0.035) | (0.032) |  | | | (0.046) | (0.05) | (0.052) | (0.05) |  | (0.074) | (0.100) | (0.093) | (0.096) |  |
| ≥ 9000 | 0.056** | 0.058** | 0.045 | 0.008 |  | | | -0.029 | -0.0446 | -0.043 | -0.046 |  | -0.018 | 0.059 | -0.002 | 0.018 |  |
|  | (0.024) | (0.027) | (0.029) | (0.026) |  | | | (0.04) | (0.045) | (0.046) | (0.046) |  | (0.056) | (0.064) | (0.067) | (0.062) |  |
| Tertiary education | 0.015 | 0.054** | 0.008 | 0.024 |  | | | -0.003 | -0.006 | -0.051 | 0.003 |  | 0.065 | 0.0449 | -0.05 | 0.055 |  |
|  | (0.019) | (0.021) | (0.023) | (0.021) |  | | | (0.032) | (0.035) | (0.037) | (0.036) |  | (0.05) | (0.062) | (0.062) | (0.06) |  |
| Number of doctor visits | 0.004* | 0.007*** | 0.007*** | 0.005** |  | | | 0.002 | 0.007** | 0.008*** | 0.001** |  | -0.01* | -0.001 | -0.004 | -0.004 |  |
|  | (0.002) | (0.002) | (0.002) | (0.002) |  | | | (0.003) | (0.003) | (0.003) | (0.003) |  | (0.005) | (0.005) | (0.06) | (0.004) |  |
| Financial risk | 0.065*** | 0.052*** | 0.049*** | 0.0105 |  | | | 0.069*** | 0.055*** | 0.062*** | 0.022 |  | 0.071*** | 0.026 | 0.072*** | 0.046** |  |
|  | (0.009) | (0.01) | (0.011) | (0.01) |  | | | (0.014) | (0.016) | (0.016) | (0.015) |  | (0.019) | (0.023) | (0.025) | (0.023) |  |
| Time preference | -0.005 | -0.019* | -0.016 | 0.009 |  | | | -0.012 | -0.007 | -0.003 | 0.021 |  | -0.071*** | -0.009 | -0.063** | -0.045 |  |
|  | (0.009) | (0.01) | (0.011) | (0.01) |  | | | (0.013) | (0.01) | (0.015) | (0.015) |  | (0.025) | (0.025) | (0.028) | (0.028) |  |
|  |  |  |  |  |  | | |  |  |  |  |  |  |  |  |  |  |
| Constant | 2.647*** | 2.581*** | 2.450*** | 2.544*** |  | | | 2.398*** | 2.273*** | 2.015*** | 2.598*** |  | 2.727*** | 2.423*** | 2.553*** | 2.645*** |  |
|  | (0.058) | (0.063) | (0.068) | (0.061) |  | | | (0.161) | (0.16) | (0.172) | (0.161) |  | (0.168) | (0.183) | (0.186) | (0.16) |  |
|  |  |  |  |  |  | | |  |  |  |  |  |  |  |  |  |  |
| Observations | 3,903 | 3,903 | 3,903 | 3,903 |  | | | 1,455 | 1,455 | 1,455 | 1,455 |  | 678 | 678 | 678 | 678 |  |
| R-squared | 0.034 | 0.026 | 0.022 | 0.041 |  | | | 0.046 | 0.038 | 0.053 | 0.028 |  | 0.137 | 0.098 | 0.148 | 0.081 |  |
| Adjusted R-squared | 0.026 | 0.017 | 0.013 | 0.033 |  | | | 0.03 | 0.021 | 0.036 | 0.011 |  | 0.097 | 0.056 | 0.108 | 0.039 |  |
| Source: Swiss Health Insurance Literacy Survey 2021. Notes: The table shows the estimated coefficients from weighted multiple linear regressions per language groups for each subscale of the HIML-CH. Robust standard errors are in parenthesis. Scales: 1 corresponds to *Confidence in choosing a health plan*; 2 corresponds to *Comparing health plans*; 3 corresponds to *Confidence in using a health plan*; 4 corresponds to *Being proactive*. Robust standard errors in parentheses; *** p<0.01, ** p<0.05, * p<0.1. Other controls included 26 canton dummies. | | | | | | | | | | | | | | | | | |
